# Quantifying Quadriceps Forces during Running Performed with and without Infrapatellar Straps

**DOI:** 10.5114/jhk/190143

**Published:** 2024-12-06

**Authors:** Xueying Zhang, Weiyan Ren, Xingyue Wang, Jie Yao, Fang Pu

**Affiliations:** 1 Key Laboratory of Human Motion Analysis and Rehabilitation Technology of the Ministry of Civil Affairs, Beijing Advanced Innovation Centre for Biomedical Engineering, School of Biological Science and Medical Engineering, Beihang University, Beijing, China.; 2 Key Laboratory of Biomechanics and Mechanobiology, Ministry of Education, School of Engineering Medicine, Beihang University, Beijing, China.; 3 State Key Laboratory of Virtual Reality Technology and System, Beihang University, Beijing, China.

**Keywords:** running-related knee injury, inverse dynamic simulation, accumulated force

## Abstract

Running-related knee injuries are associated with high and repetitive quadriceps contractions. Infrapatellar straps are commonly recommended for the prevention and management of those injuries. The effects of infrapatellar straps have been investigated in terms of quadriceps activations. However, these indexes cannot accurately characterize the quadriceps forces, which directly contribute to knee injuries. This study aimed to quantify quadriceps forces during running performed with and without infrapatellar straps based on OpenSim. Experimental data from 18 healthy participants were recorded using a 10-camera motion capture system and two force plates when they performed running at self-selected speeds with and without infrapatellar straps. OpenSim was used to estimate muscle forces, muscle activity, joint kinematics, and joint kinetics. The use of infrapatellar straps significantly reduced peak quadriceps forces (p < 0.001), accumulated forces of quadriceps (p < 0.001), and peak knee extension moments (p < 0.001). Among the four distinct muscles of the quadriceps, the vastus lateralis contributed the most to the reduction in quadriceps muscle forces. Strapping did not result in a significant change in rectus femoris forces (p > 0.05). The use of infrapatellar straps results in lower vastus muscle forces, and thus could be helpful in managing and preventing running-related knee injuries. However, infrapatellar straps may have little effect in people with an excessively forceful rectus femoris.

## Introduction

Running is a popular leisure-time physical activity (Lee et al., 2017). It can provide significant health benefits in terms of preventing chronic diseases and increasing longevity (Lee et al., 2017). However, running can cause injuries to the lower extremities. A study found that the incidence of lower extremity running-related injuries was up to 79% (Van Gent et al., 2007). The knee is the predominant injury site, accounting for 50% of lower extremity injuries, with a significant percentage arising from conditions, such as patellar tendinopathy (PT), patellofemoral pain syndrome (PFPS), and Osgood-Schlatter disease (OSD) (Rosen et al., 2017; Van Gent et al., 2007). Therefore, a widespread concern has been raised for the prevention and treatment of running-related knee injuries.

The infrapatellar strap (IS) is commonly recommended to prevent running-related knee injuries by clinicians during physical activity because they are affordable, nonrestrictive, and noninvasive, and can fit any knee (Rosen et al., 2017; Straub and Cipriani, 2012). For running-related knee injuries, the quadriceps is the muscle of focus because these injuries are typically caused by high, repeated, and continuous traction from the quadriceps muscle (Bek et al., 2011; Dar and Mei-Dan, 2019; Enomoto et al., 2019; Lavagnino et al., 2011). A study by Rosen et al. (2017) using surface electromyography (EMG) concluded that patellar tendon straps reduced quadriceps activation before drop-jump landing, particularly in the vastus lateralis (VL). The authors speculated that these changes might decrease tensile stress on the patellar tendon. In Straub and Cipriani's (2012) study, they failed to detect any differences in normalized mean or peak EMG activity in any part of the quadriceps during a body-weight squat with and without infrapatellar straps. These studies have solely used EMG to find differences in quadriceps muscle activity due to strapping during dynamic and static movements such as landing and squatting. Moreover, the results obtained from these studies were inconsistent. Excessive and repetitive loading of quadriceps muscle has been shown to contribute to the symptoms associated with running-related knee injuries (Bek et al., 2011; Enomoto et al., 2019; Lavagnino et al., 2011). However, fewer studies have directly investigated changes in quadriceps muscle forces with infrapatellar straps during dynamic running tasks.

The purpose of this study was to quantify quadriceps forces during running performed with and without infrapatellar straps in asymptomatic participants using OpenSim. To clarify the indications and principles of rehabilitation with ISs, we also calculated the muscle forces of particular muscles of the quadriceps muscle, the accumulated forces of the quadriceps muscle, the activity of particular quadriceps muscles, as well as knee joint angles and moments during running. We hypothesized that ISs would reduce quadriceps forces during running, with a different response in the quadriceps muscle heads.

## Methods

### Participants

Eighteen healthy subjects participated in this study (12 males and 6 females). The mean (± SD) age, body height, and body mass of participants were 25.44 ± 2.62 years, 1.70 ± 0.08 m, and 67.51 ± 11.58 kg, respectively. All participants were right-leg dominant (the leg most often used for kicking a ball) and recreationally active university student volunteers (participated in 90 minutes or more of physical activity per week). Participants were excluded based on the following criteria: (1) musculoskeletal injury within the previous six months, (2) history of recurrent knee pain, and (3) previous surgery on the lower extremities. The study was approved by the Science and Ethics Committee of the Beihang University (protocol code BM20200067; approval date: 26 March 2020), and written informed consent was obtained from all participants included in the study.

### Experimental Protocol

**Figure 1:**
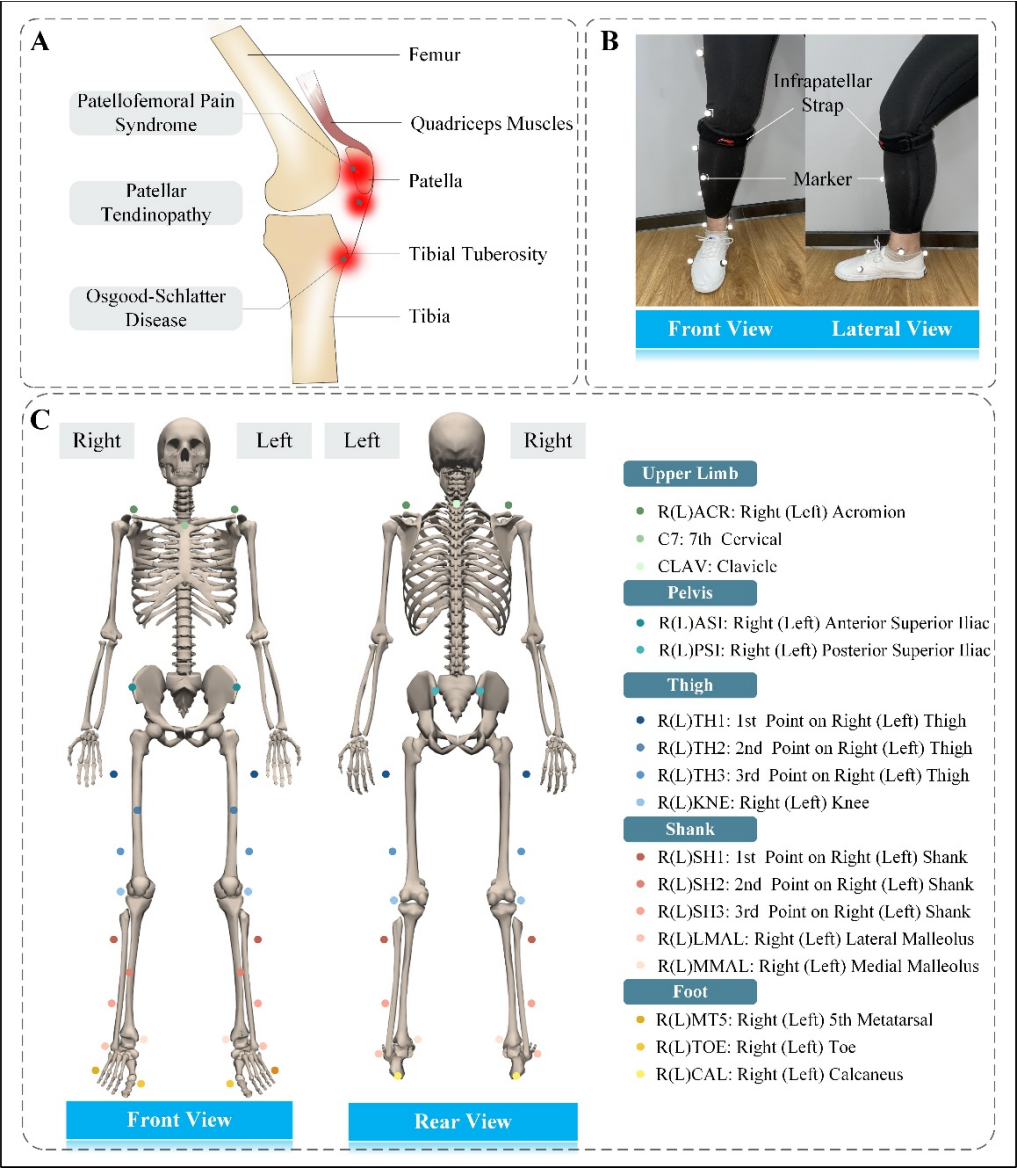
Common sites of a few running-related anterior knee injuries and the experimental setup. (A) Lateral view of the knee joint. Red areas represent the common sites for patellofemoral pain syndrome, patellar tendinopathy, and Osgood-Schlatter disease. (B) Participants wearing the infrapatellar straps. (C) Position of the Vicon markers in front and back views (skeleton from OpenSim). A total of 32 markers were placed, and the locations were the shoulder, C7 vertebra, clavicle, anterior and posterior superior iliac, thighs, knees, shanks, and feet.

Participants were instructed to run under two strapping conditions (with and without ISs) in random order. For the strapping session, participants were fitted with commercial ISs (AQAH236-1; Li-Ning Inc., Beijing, China) on both legs. According to the manufacturer's instructions and the most common site of running-related knee injuries (Figure 1A), the IS encircled the leg just below the patella, and the rubber tubular member was pressed on the patellar tendon (Figure 1B). The same investigator set up all ISs. All participants wore the same type of suitably sized clothing and sneakers to reduce side effects (Figure 1B).

A total of 32 infrared-reflecting markers were mounted on the torso, thighs, shanks, and feet to track the movement of participants (Figure 1C). Three-dimensional kinematic data were collected using a ten-camera optical motion capture system (Vicon MX; Oxford Metrics, UK) at a sampling frequency of 100 Hz. All cameras were fixed to the walls around the laboratory to avoid vibration interference. The ground reaction forces (GRFs) were recorded at a sampling rate of 1000 Hz using two force plates (AMTI; Watertown, MA, USA) embedded into a 10-meter walkway. All data were synchronized using Vicon Workstation v2.6 software. The kinematic and GRF data were low-pass filtered (15 Hz) using a zero-lag fourth-order Butterworth filter (Maniar et al., 2020).

**Figure 2:**
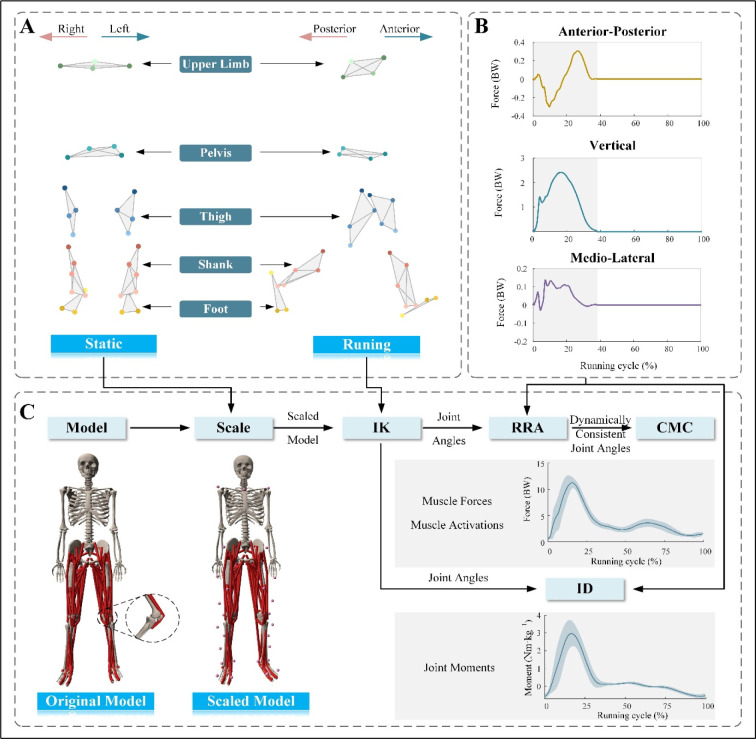
Flowchart of the experiment and inverse dynamic analysis. (A) Visualization of the static marker trajectory (left) and the dynamic marker trajectory (right). The static marker trajectory was used to scale the generic model's segments to each participant's anthropometry. (B) Normalized vertical, anterior-posterior, and medio-lateral GRFs in one sample trial. The stance phase (gray shading) was defined as the period between the foot's initial contact and toe-off in a running cycle. (C) The flowchart for inverse dynamic analysis of each participant's motion with OpenSim. The muscle force and joint moment are normalized to the body weight and body mass, respectively. BW: body weight

Prior to data collection, participants performed a 5-min warm-up of running on the overground to familiarize themselves with the experimental environment. Then, participants performed three practice trials along the walkway to find their self-selected speed and to determine the starting position for each trial. The self-selected running speed, at which participants could run comfortably, was determined using the average running speed of these three trials. The speed was monitored using a pair of lights. The lights were positioned before and after the force plates. The distance between the two lights was 4 m. The lights were placed at shoulder height of each participant to avoid being affected by an arm swing. Participants were instructed to reach their self-selected speed before entering the speed record volume and to maintain that velocity throughout the recording. Participants were asked to focus on an object on the wall in front of them while running to avoid targeting the force plates (Queen et al., 2006). Targeting the force plates may result in stride adjustment and thus may affect the measurement ( Abendroth-Smith, 1996). After each running trial, participants walked back to the starting point and were allowed to rest if needed between trials to avoid fatigue. For each participant, a static trial with the participant standing in a neutral upright stance was performed for model adaptation (Figure 2A). Subsequently, five valid trials were recorded for each participant with and without straps (Figures 2A and B). A trial was considered valid if (1) the running speed was within ± 5% of the self-selected speed, (2) the force plates were not targeted by participants and were not missed, and (3) there was no loss of balance. A 10-min rest interval was provided after the completion of the five valid trials for one condition.

### Musculoskeletal Simulation

A full-body musculoskeletal model (Rajagopal et al., 2016) was used in the present study to perform simulations in OpenSim 4.2 (Simbios, Stanford, CA, USA) (Delp et al., 2007) (Figure 2C). The model had 37 degrees of freedom (20 degrees of freedom in the lower body and 17 in the torso and the upper body). Further, there were 80 muscle-tendon units in the lower body (40 per leg) and 17 torque actuators in the upper body (one for each degree of freedom in the upper body) (Rajagopal et al., 2016).

A standard pipeline was used to perform the musculoskeletal simulations (Figure 2C). First, the Rajagopal model was scaled to match each participant anthropometrically using static trial data. Second, the inverse kinematics algorithm was used to estimate the knee flexion angles. Third, knee extension moments were calculated using the inverse dynamics tool. Subsequently, the dynamically consistent alignment between the GRFs and model kinematics was achieved using a residual reduction algorithm. The computed muscle control algorithm was employed to calculate the muscle forces and activity of the VL, the vastus medialis (VM), the vastus intermedius (VI), and the rectus femoris (RF), as well as the quadriceps muscle forces (sum of the forces of the VL, VM, VI, and RF) and hamstring muscle forces (sum of the forces of the long head of the biceps femoris, short head of the biceps femoris, semimembranosus, and semitendinosus muscles).

### Parameter Computation

Five running cycles per condition for each participant were averaged. The dominant leg was used for analysis because biomechanical factors associated with a higher risk of knee injuries were observed in the dominant leg compared to the non-dominant one, such as greater contracted power during the propulsion phase of running and higher knee extension isometric torque (Hunter et al., 2000; Wang et al., 1993). Based on the GRF vertical component, initial foot contact, toe-off, the stance phase, and the swing phase were determined. The running cycle was defined as the period from initial contact of one foot until the initial contact of the same foot (Novacheck, 1998). The initial foot contact and the toe-off indicated the start of the stance and the swing phase, respectively (Clark et al., 2023; Novacheck, 1998). The kinematic, dynamic, and muscle force data of the time series were interpolated to the same length before being averaged. The muscle force and joint moment were normalized to the body weight (BW) and body mass (Nm•kg^−1^) of each participant, respectively. The continued tension of the quadriceps and hamstring muscles was represented as accumulated force of quadriceps and hamstring muscles. The accumulated force was defined as the area under the force-time curve throughout a running cycle, and it was expressed as follows:


Acc=∫Fdt#

where *Acc* indicated the accumulated force, *F* represented the muscle force, and *t* designated the time. The accumulation of hamstring muscles was calculated to explore the effect of ISs on the protagonist-antagonist muscle balance.

### Validation of Simulation

To evaluate the reliability of the simulations, the simulated muscle activity of the no-strap group was compared with EMG data from the literature (Hamner and Delp, 2013). The EMG data from ten healthy participants, who ran at a speed of 3 m/s, were recorded in their study. The mean age, body height, and body mass of participants were 29 years, 1.77 m, and 70.9 kg, respectively. The magnitude of the correlation between EMG and simulated muscle activity in the full running cycle and the timing of correlation between peak EMG and peak simulated activity were calculated using statistical software SPSS 26 (IBM Corporation, NY, USA). The major lower extremity muscles used to calculate the correlation were the VL, the VM, the soleus, the RF, the tibialis anterior, the gluteus maximus, and the gluteus medius. The Spearman correlation factor was calculated, with the *r*-value indicating the strength of the linear relationship between the variables.

### Statistical Analysis

The analyzed variables, including running speeds, peak quadriceps muscle forces, quadriceps muscle forces and activity over a running cycle, peak knee flexion angles, peak knee extension moments, as well as accumulated forces of the quadriceps and hamstring muscles, were tested for normal distribution using the Shapiro-Wilk test. For continuous variables over time, specifically the quadriceps muscle forces and activity, paired statistical parametric mapping (SPM) *t*-tests (Pataky, 2010) were used. The SPM analysis was conducted using open-source SPM1D package (https://www.spm1d.org) in MATLAB (The MathWorks, Inc., Natick, Massachusetts, USA). The running speeds, accumulated forces of the quadriceps and hamstring muscles, peak knee flexion angles, and peak knee extension moments between the with and without the strap conditions were compared using the paired *t*-test because the data were normally distributed. For non-normally distributed data, the peak quadriceps muscle forces between the two conditions were compared using the Wilcoxon signed-rank test. Data were statistically analyzed using SPSS. The reported statistical significance levels were all two-sided, and a *p*-value of less than 0.05 was deemed statistically significant. Effect sizes using Cohen's *d* were also calculated for each of the comparisons and the following thresholds were used: 0.20 = small, 0.50 = moderate, 0.80 = large (Cohen, 1992).

## Results

**Figure 3:**
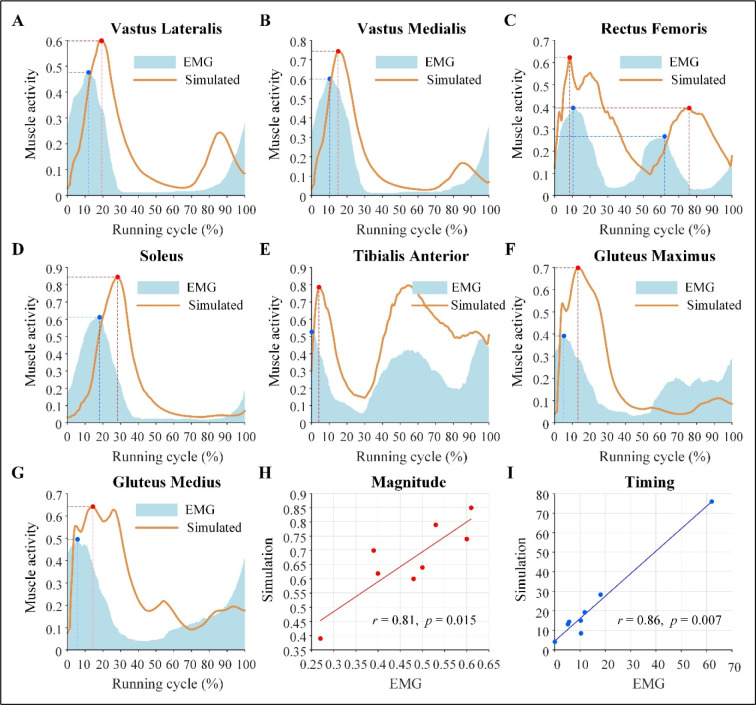
Simulated muscle activity and measured EMG curves along with the magnitude and timing correlations between simulated muscle activity and EMG. (A–G) Simulated muscle activity and measured EMG curves during a full running cycle for the vastus lateralis, vastus medialis, rectus femoris, soleus, tibialis anterior, gluteus maximus, and gluteus medius muscles, respectively. (H) Magnitude correlation between simulated muscle activity and EMG. (I) Timing correlation between peak simulated activity and peak EMG.

As shown in Figures 3A–G, the simulated muscle activity and EMG data showed similar features. The magnitude of activity was significantly correlated with that of the EMG (*r* = 0.81, *p* = 0.015) (Figure 3H). The timing of peak activity was delayed compared to EMG, but was significantly correlated (*r* = 0.86, *p* = 0.007) (Figure 3I).

Running speeds were similar under unstrapped (mean ± SD, 2.91 ± 0.34 m/s) and strapped (2.92 ± 0.29 m/s) conditions (*p* = 0.713) (Table 1). The peak quadriceps muscle forces when using ISs (9.29 ± 1.74 BW) were significantly lower (*p* < 0.001) than when using no strap (11.11 ± 2.04 BW), with a large effect size (*d* = 2.10). The paired *t*-test revealed significant differences in the accumulated forces of quadriceps (no-strap: 1825.94 ± 327.42 Ns, strap: 1650.65 ± 297.57 Ns, *p* < 0.001) and hamstring (no-strap: 737.66 ± 110.52 Ns, strap: 706.59 ± 114.90 Ns, *p* = 0.018) muscles across all participants. Specifically, with the strap, participants displayed lower accumulated forces with a large (*d* = 1.73) and a moderate to large (*d* = 0.61) effect size, respectively (Table 1).

**Table 1:** Velocity, force, and accumulated force of quadriceps and hamstring muscles, and joint angle and moment of the knee in the sagittal plane.

	No brace	Infrapatellar strap	*p*-value	Cohen's *d*
Running speed (m/s)				
	2.91 ± 0.34	2.92 ± 0.29	0.713	−0.09
Peak force (BW) developed by quadricep muscle force				
	11.11 ± 2.04	9.29 ± 1.74	< 0.001	2.10
Accumulated force (Ns) developed by quadriceps and hamstring muscle force				
Quadriceps	1825.94 ± 327.42	1650.65 ± 297.57	< 0.001	1.73
Hamstring	737.66 ± 110.52	706.59 ± 114.90	0.018	0.61
Peak knee flexion angle (°)				
	90.82 ± 12.22	93.04 ± 10.69	0.104	−0.41
Peak knee extension moment (Nm•kg^−1^)				
	3.23 ± 0.71	2.88 ± 0.71	< 0.001	1.18

Data are means ± SD. BW: body weight

**Figure 4:**
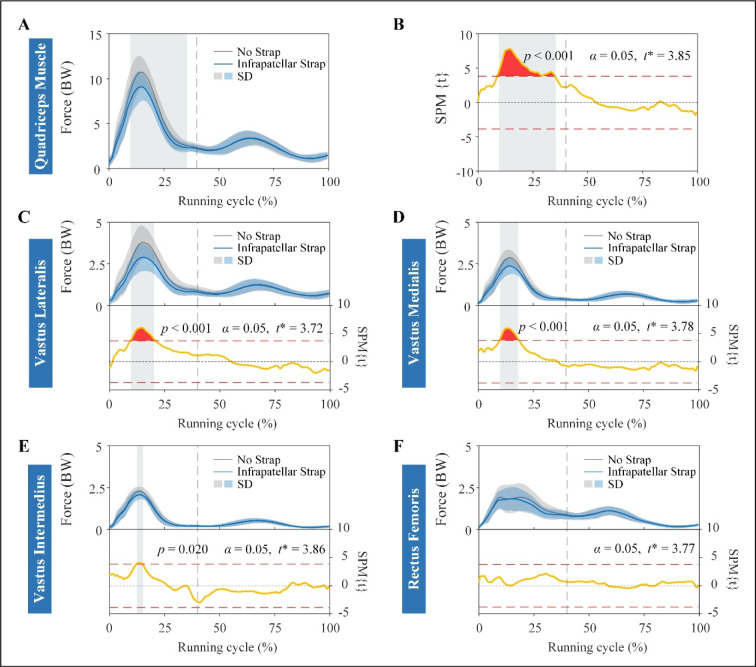
Muscle forces and results of SPM curves of quadriceps and individual muscles of quadriceps. Muscle forces (A) and results of SPM{t} curves (B) of the quadriceps muscle. Force (top) and SPM{t} (bottom) curves of vastus lateralis (C), vastus medialis (D), vastus intermedius (E), and rectus femoris (F) muscles. The force is described as the mean (solid line) ± standard deviation (shaded area), and it is normalized by the body weight of each participant. The shaded areas on the SPM{t} curve show the track locations where the SPM{t} curve exceeded the critical threshold (red dotted line). The *p*-values are provided for these suprathreshold clusters. Gray dot lines refer to the moment of toe-off. BW: body weight

The SPM results showed that quadriceps muscle forces were significantly lower during the stance phase of the running cycle (between 9% and 35.5%) when using ISs compared to the no strap condition, where it exceeded the critical *t*-statistic threshold at *p* < 0.001 (Figures 4A and B). Among the four muscles that comprise the quadriceps, the VL, VM, and VI muscle forces decreased significantly (*p* < 0.001, *p* < 0.001, and *p* = 0.02, respectively) during eccentric quadriceps contraction in the stance phase (between 10% and 21%, 10.5% and 19%, and 13% and 15.5% of the running cycle, respectively) with the use of ISs (Figures 4C–E). No significant difference was observed in the RF forces between the two strapping conditions in the full running cycle (Figure 4F).

**Figure 5:**
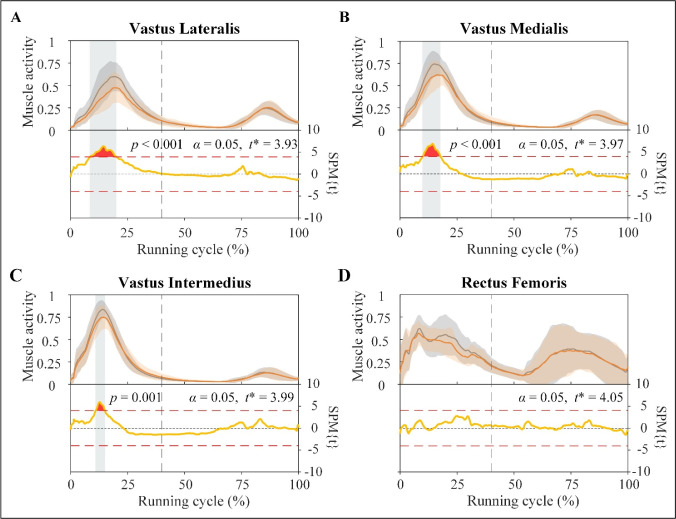
Activity (top) and results of SPM curves (bottom) of vastus lateralis (A), vastus medialis (B), vastus intermedius (C), and rectus femoris (D) muscles. The activity is described as the mean (solid line) ± standard deviation. The shaded areas on the SPM{t} curve show the track locations where the SPM{t} curve exceeded the critical threshold (red dotted line). The *p*-values are provided for these suprathreshold clusters. Gray dot lines refer to the moment of toe-off.

The VL, VM, and VI muscle activity decreased significantly (*p* < 0.001, *p* < 0.001, and *p* = 0.001, respectively) during the stance phase (between 8.6% and 20.2%, 10% and 17.8%, and 11% and 15.1% of the running cycle, respectively) with the use of ISs. However, the use of ISs when running slightly altered the activity of the RF (Figure 5).

The results of the joint kinematics and kinetics are presented in Table 1. ISs did not change the peak knee flexion angles significantly (no-strap: 90.82 ± 12.22, strap: 93.04 ± 10.69, *p* = 0.104). A significant difference in the peak knee extension moment between wearing ISs and not wearing ISs was found (*p* < 0.001) and had a large effect size (*d* = 1.18). The peak knee extension moments were lower while wearing straps (2.88 ± 0.71 Nm•kg^−1^) than while not wearing straps (3.23 ± 0.71 Nm•kg^−1^).

## Discussion

This study investigated the immediate effects of ISs on quadriceps forces during running in asymptomatic participants. To the authors' knowledge, only few biomechanical studies have examined how an IS affects quadriceps forces during dynamic movement. The results showed that ISs were associated with a significant reduction in the peak and accumulated forces of the quadriceps muscles, especially the vastus muscles. However, strapping did not result in a significant change in the RF forces. These results suggest that ISs potentially benefit running-related knee injuries by reducing vastus loading. However, individuals who need to reduce the excessive RF forces may experience little benefit from wearing straps.

In this study, the reliability of muscle force calculation was examined by comparing simulated muscle activity with EMG data. As a noninvasive validation method, EMG can provide direct and indirect information about the electrical activity of muscles (Karimi et al., 2021) and is frequently used to measure muscle activity ( Disselhorst-Klug et al., 2009; Heintz and Gutierrez-Farewik, 2007). Therefore, the simulated muscle activity in this study was compared with the EMG data published by Hamner et al. (2013). In the study by Hamner et al. (2013) the running speed was similar to that in the present study (3 m/s versus 2.91 m/s). Moreover, the delay between the EMG and muscle activity was consistent with the electromechanical delay reported by Corcos et al. (1992). Electromechanical delays comprise the transport time and the time required to generate detectable changes in force (Corcos et al., 1992). The results of the present study provide the validity of the muscle force calculation.

ISs not only reduced the peak quadriceps forces, but also reduced the accumulation of quadriceps forces over the running cycle. As previously mentioned, high, repeated, and continuous traction from the quadriceps muscle is the reason for running-related knee injuries, such as OSD, PFPS, and PT (Bek et al., 2011; de Vries et al., 2016; Enomoto et al., 2019), indicating that not only the peak quadriceps forces, but also the periodic accumulation of quadriceps forces should be considered. Despite this, previous studies have mainly focused on the quadriceps force itself, without considering its accumulation over the running cycle. The results of the present study suggest that ISs significantly reduce high and continuous traction, which may be beneficial for the management and prevention of running-related knee injuries.

The findings of the present study support our hypothesis that the response in muscle forces is different between the muscle heads of the quadriceps. A reduction in muscle forces was observed in the vastus muscle, but not in the RF muscle head, which was consistent with the variation in the corresponding muscle activities. The VL is the largest muscle of the quadriceps and has a high force-generating potential, and it operates in concert with the tendon (Bohm et al., 2021). Therefore, the pressure exerted on the patellar tendon through ISs appeared to have a greater effect on the VL. The VM and the VI operate similarly to the VL since they share the same single patellar tendon. Thus, the responses of the VM and the VI to the application of the IS were consistent with those of the VL. The electrophysiological activity of a muscle initiates force production; hence, the higher the EMG level, the greater the forces developed by the muscle (Disselhorst-Klug et al., 2009). The reduction in vastus forces during the stance phase with ISs was due to the lower muscle activity during this phase. This lower muscle activity may be explained by (i) fewer fibers being activated during the eccentric quadriceps contraction phase, (ii) external pressure changing the length of the vastus muscles and allowing for more efficient contraction, and (iii) other motor units within the vastus muscles or other muscles offering a greater contribution to running (Earl et al., 2004; Snyder-Mackler and Epler, 1989). The results suggest that ISs could be effective for individuals who particularly need to reduce excessive vastus forces, and individuals with knee injuries caused by excessive RF forces may experience little benefit from wearing ISs; however, further research is needed, particularly in symptomatic participants.

The accumulated forces of the hamstring muscle decreased significantly when wearing ISs. As agonist and antagonist muscles, close collaboration between the quadriceps and hamstring muscles is integral to maintaining knee joint stability during motion (Wu et al., 2017). Strength imbalances in the agonist and antagonist muscles may increase the risk of injury to the lower extremity (Lutz et al., 2023). Hence, ISs can reduce the accumulated forces of quadriceps muscle without major alterations in the protagonist-antagonist muscle balance, which is essential for the effective application of ISs. Moreover, decreased quadriceps and hamstring accumulated forces may affect the energetics at the lower extremity joints during running, thereby decreasing energy costs.

Wearing ISs significantly reduced the peak knee extension moment in this study. The quadriceps are the major contributors to the knee extension moment (Earl et al., 2004), and the VL is the largest quadricep muscle and has a high force-generating potential during running (Bohm et al., 2021). In our study, the decreased activity of the VL, the VM, and the VI, especially the VL, was noted in the stance phase of a running cycle. The reduced peak knee extension moment was also observed in the stance phase of the running cycle. Thus, reduced vastus muscle activity may be the main reason for the reduction in the peak knee extension moment in the present study. However, ISs worn during running had no significant effect on the peak knee flexion angles compared with not wearing ISs, implying that ISs do not limit knee movement when used while running. Similar results were noted by Sinclair et al. (2017). They reported that knee braces did not significantly hinder the sagittal plane movement of the knee in professional athletes wearing knee sleeves during running ( Sinclair et al., 2017). The decreased peak knee moment and similar peak knee angles indicate that ISs may help in reducing tensile stress of the knee extensor mechanism and in alleviating running-related knee injuries caused by the abnormal knee extensor mechanism without hindering joint function.

This study had a few limitations. First, only a healthy population was selected for experiment participants. This study aimed to investigate the changes in quadriceps muscle forces between two strapping conditions instead of the absolute values of the loads. The results provided baseline data that will be beneficial for future studies in the symptomatic population that should be considered in future studies. Second, only young adults were enrolled. PT and PFP are common complaints in active adults, whereas OSD is typically observed in adolescent athletes. However, OSD-related symptoms can persist into adulthood and affect the daily life and sports activities of young adults. Future research is necessary to observe the changes in ISs in older and younger participants. Third, PT is more common among athletes in jumping sports (Dar and Mei-Dan, 2019), while the current focus was on running. Future studies should investigate the effect of ISs on quadriceps forces when performing jumping. Additionally, only the quadriceps muscle activity was analyzed. Other lower extremity muscles also contributed to running, which may partly explain the similarity in speed under the two conditions. Future studies should also investigate the effects of ISs on the activity of other lower limb muscles.

## Conclusions

In conclusion, all the participants demonstrated lower peak and accumulated forces in the quadriceps muscle when running with ISs. The significant decrease in accumulated forces was strongly associated with a reduction in the vastus muscle forces during the stance phase. This indicates that ISs provide some protection for the knee and could be used as a conservative treatment and prevention measure for running-related knee injuries, especially for individuals who need to reduce excessive vastus muscle forces. This study provides initial evidence regarding the effectiveness of ISs on quadriceps force reduction and helps clarify the indications and principles of rehabilitation with ISs. This is an important step in the use of ISs in symptomatic populations.
